# Hepatic HSD17B6 is dispensable for diet-induced fatty liver disease in mice

**DOI:** 10.1016/j.bbrep.2025.101924

**Published:** 2025-01-19

**Authors:** Delong Yuan, Nan Bai, Qihan Zhu, Shaoxuan Song, Anyuan He, Jianqing Wang, Yali Chen

**Affiliations:** a Department of Biochemistry and Molecular Biology, College of Life Sciences, Anhui Medical University, Hefei, 230032, Anhui, China; b Department of Pharmacy, Anhui Medical University, Hefei, 230032, Anhui, China; c Department of Biochemistry and Molecular Biology, College of Basic Medical Science, Anhui Medical University, Hefei, 230032, Anhui, China

**Keywords:** MAFLD, HSD17B6, Fatty liver, SREBP

## Abstract

Metabolic dysfunction-associated fatty liver disease (MAFLD) affects up to a third of the global population, which causes huge both clinical and economic burdens. However, its therapeutic strategy is still limited. Steroid dysregulation plays a pivotal role in the homeostasis of lipid metabolism. 17-beta-hydroxysteroid dehydrogenase type 6 (HSD17B6)—one member of 17β-HSDs, encoded by the gene *Hsd17b6*, catalyzes the synthesis of androsterone and estrone—steroid hormones. However, whether the manipulation of HSD17B6 could ameliorate diet-induced fatty liver disease remains unknown. Here, we found that the expression of *Hsd17b6* is enriched in the liver in both humans and mice. The data of single-cell RNA-seq suggests that *Hsd17b6* appears to be exclusively expressed in hepatocytes—the parenchymal cells of the liver. Furthermore, the hepatic expression of *Hsd17b6* is correlated with fatty liver disease. A mouse model with *Hsd17b6* deletion in the liver (HLKO) is successfully generated via the administration of AAV8 expressing Cre recombinase (driven by TBG—a liver-specific promoter) and sgRNAs of *Hsd17b6* to Cre-dependent Cas9 mice. Control and HLKO mice were challenged with the high-fat choline-deficient diet—a diet widely used for the model generation of fatty liver disease. Interestingly, the HLKO liver shows a special proteome signature, with the altered proteins enriched in the Golgi apparatus. However, the deletion of *Hsd17b6* does not affect fatty liver disease in terms of fat accumulation, inflammation, and hepatic fibrosis. Taken together, our study suggests that the expression of *Hsd17b6* is enriched in the liver and correlated with fatty liver disease but its hepatic deletion does not affect diet-induced fatty liver disease.

## Highlights

• Hsd17b6 is exclusively expressed in hepatocytes—the parenchymal cells of the liver.
• The hepatic expression of Hsd17b6 is associated with fatty liver disease.
• Hepatic Hsd17b6 is dispensable for diet-induced fatty liver disease.

## 1 Introduction

The term MAFLD, metabolic dysfunction-associated fatty liver disease, encompasses all fatty liver disease states and represents a spectrum of liver disease from “benign” steatosis, steatohepatitis, cirrhosis, and hepatocellular carcinoma (HCC) [1]. MAFLD affects up to a third of the global population [1]. Although MAFLD causes huge both clinical and economic burdens, its therapeutic strategy is still limited. Therefore, further studies are required to decode the mechanism underlying the progression of MAFLD and to discover new therapeutic targets.

17-β-Hydroxysteroid dehydrogenases (17β-HSDs) are oxidoreductases that play essential roles in estrogen and androgen steroid metabolism by catalyzing the final steps of steroid biosynthesis. Fourteen different subtypes have been identified in mammals, which catalyze NAD(P)H or NAD(P) (+) dependent reductions/oxidations at the 17-position of the steroid [2]. Considering the pivotal role of 17 β-HSDs in steroid hormone metabolism and their substrate specificity, these enzymes are promising therapeutic targets for diseases such as liver cancer, breast cancer, and prostate cancer.

17-β-hydroxysteroid dehydrogenase type 6 (HSD17B6)—one member of 17β-HSDs, encoded by the gene *Hsd17b6*, converts 5-alpha-androstan-3-alpha, 17-beta-diol to androsterone and estradiol to estrone [3]. Sterol regulatory element-binding proteins (SREBPs) are master regulators of lipid metabolism via the sensing of sterol and insulin. Of note, it has been reported that HSD17B6 inhibits SREBP signaling by binding with SREBPs [4]. Intriguingly, the enzymatic activity of HSD17B6 is not required by its inhibitory effect on SREBP signaling, indicating it may also regulate lipid metabolism in a manner independent of its synthetic activity of steroid hormones.

Interestingly, the expression level of *Hsd17b6* is associated with clinical prognosis of hepatocellular carcinoma—the late stage of MAFLD [5]. Meanwhile, *Hsd17b6* appears to be highly expressed in liver of humans [5]. Therefore, we propose a hypothesis that the manipulation of hepatic HSD17B6 might ameliorate MAFLD. To test this hypothesis, the mouse model with hepatic deletion of *Hsd17b6* was generated and fed with the high-fat choline-deficient diet (HFCDA) to assess its effect on fatty liver disease in this study.

## 2 Materials and methods

### 2.1 Animal experiment

All animal experiments were conducted in accordance with the National Institutes of Health guide for the care and use of Laboratory animals (NH Publications No. 8023 revised 1978) and were approved by the Animal Ethics Committees at Anhui Medical University (Permission no. LLSC20210007. March 1, 2021). Experimental findings were reported based On ARRIVE guidelines. The mice, including Cre-dependent lox-stop-lox spCas9 mice and wild-type mice on C57BL/6J genetic background, had free access to water and were fed with a normal chow diet, or the high-fat choline-deficient diet (Research diet, A06071302) as indicated in figure legends. Adeno-associated virus serotype 8 was packaged and administrated as previously reported [6]. More detailed information was provided in Supplementary method.

### 2.2 Real-time quantitative PCR

Quantitative PCR was performed as previously [7,8]. The primers used are listed in Supplementary Table 1.

### 2.3 Western blotting

Western blotting for total lysate of livers was performed as previously [8]. The antibodies used in this study are as follows: ACTA2 (Zenbio, 380653), Col1a1 (Zenbio, 501352), SREBP1 (Proteintech, 14088-1-AP), SCAP (Proteintech, 12266-1-AP), and Tubulin (Beyotime, 11224-1-AP).

### 2.4 Biochemical assays

Serum triglyceride (A110-1-1, Jiancheng), total cholesterol (A111-1-1, Jiancheng), alanine aminotransferase (ALT) (C009-2-1, Jiancheng), and estrone (EU3107, Finetest) were measured according to the manufacturer’s instructions.

### 2.5 Protein mass spectrometry

The Astral DIA-based proteomic analysis was conducted by MajorBio (Shanghai, China). Briefly, the proteins were extracted and digested with Trypsin. After trypsin digestion, the peptides were drained by a vacuum pump. Then, the enzymatically drained peptides were re-solubilized with 0.1 % trifluoroacetic acid, and the peptides were desalted with a hydrophilic-lipophilic balance column and drained by a vacuum concentrator. Finally, the peptides were quantified using the Thermo Fisher Scientific Peptide Quantification Kit (item #23275). Based on peptide quantification results, the peptides were analyzed by a VanquishNeo coupled with an Orbitrap Astral mass spectrometer (Thermo, USA). Data-independent acquisition (DIA) data were acquired using an Orbitrap Astral mass spectrometer operated in DIA mode. MS data were collected over a *m/z* range of 100 to 1700. Spectronaut software (Version 18) was used to search the DIA raw data. 6 peptides per protein and 3 daughter ions per peptide were selected for quantitative analysis. Differently expressed proteins (DEPs) (FC > 2.0, p < 0.05) were subjected to GO enrichment analysis using the online software DAVID (https://david.ncifcrf.gov/) with the default setting. The mass spectrometry proteomics data have been deposited to the ProteomeXchange Consortium (PXD056581).

### 2.6 Histology

Oil Red O staining was performed with a standard protocol. Briefly, the liver was dissected and fixed in 10 % formalin, equilibrated in 20 % sucrose, embedded in OCT compound, and cut into sections with 7-μm thickness. The sections were fixed in 10 % formalin, stained with 0.5 % Oil Red O, and examined under a microscope.

### 2.7 Statistics

Data are reported as mean ± standard error of the mean (SEM) unless stated otherwise. Statistical analysis and graphs were generated using GraphPad Prism software. Statistical comparisons between two groups were performed by using the unpaired student t-test. The significance was defined as ∗*p* < 0.05, ∗∗*p* < 0.01, ∗∗∗*p* < 0.001.

## 3 Results and discussions

### 3.1 The expression of Hsd17b6 is enriched in liver and associated with metabolic dysfunction-associated fatty liver disease

**Fig. 1:**
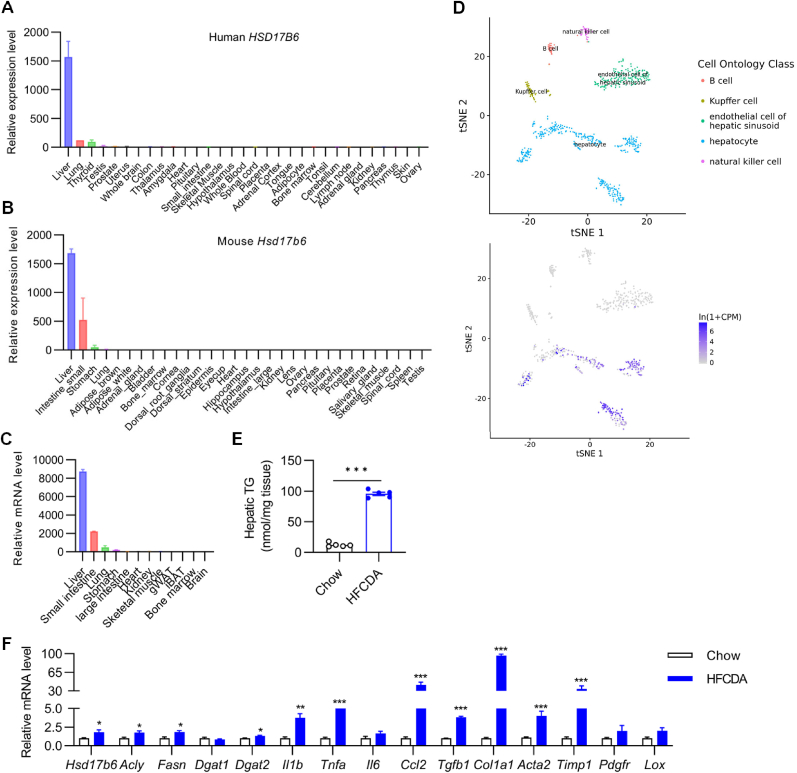
The expression of *Hsd17b6* is enriched in the liver and associated with metabolic dysfunction-associated fatty liver disease. (A) The expression profile of human *Hsd17b6* in 30 tissues (Data from BioGPS, n = 2/tissue). (B) The expression profile of mouse *Hsd17b6* in 30 tissues (Data from BioGPS, n = 2/tissue). (C) The tissue distribution of *Hsd17b6* in 9 tissues of male C57 mice (n = 2/tissue). L32 was used as an internal control. (D) The cell ontology class (top) and the *Hsd17b6* expression profile (bottom) in mice liver (Data from Tabula Muris, https://tabula-muris.ds.czbiohub.org/). (E–F) Two groups of 8-week-old male C57 mice were fed either chow or a high-fat choline-deficient diet (HFCDA) for 3 months and sacrificed for liver collection after overnight fasting (n = 5/group). (E) Hepatic triglyceride (TG) content. (F) Hepatic expression of lipid synthetic genes (*Acly, Fasn, Dgat1,* and *Dgat2*), inflammatory markers (*Il1b*, *Tnfa*, *Il6*, *Ccl2*, and *Tgfb1*), and fibrotic markers (*Col1a*, *Acta2*, *Timp1*, *Pdgfr*, and *Lox*) assayed by qPCR. Rab1b was used as an internal control. Data were expressed as mean ± SEM and analyzed by Student’s t-test. ∗*p* < 0.05, ∗∗*p* < 0.01, ∗∗∗*p* < 0.001.

HSD17B6, encoded by the gene *Hsd17b6*, plays a role in the metabolism of steroids—a type of lipid regulating metabolism homeostasis. However, the physiological function of HSD17B6 remains poorly understood. To explore the physiological role of HSD17B6, we first examined its expression profile in human tissues by mining the BioGPS database [9]. We found that the highest expression of *Hsd17b6* is observed in the liver, followed by lung, thyroid, testis, and prostate, and is nearly undetectable in the rest of the tissues (Fig. 1A), in line with the finding of Lv et al. [5]. Similarly, the highest expression of Hsd17b6 is also found in the liver of mice, followed by the small intestine and stomach (Fig. 1B). To further confirm the expression profile, 12 tissues were collected from C57 mice and subjected to qPCR analysis. The results show a similar expression profile as the data mined from BioGPS (Fig. 1C), with the highest expression in the liver followed by the small intestine, lung, and stomach. Interestingly, *Hsd17b6* appears to be exclusively expressed in hepatocytes—the parenchymal cells of the liver (Fig. 1D). In summary, *Hsd17b6* is enriched in the liver both in humans and mice, indicating it might play a critical role in the physiology of the liver.

The dysregulation of steroid metabolism is highly correlated with fatty liver disease [10]. We are wondering whether liver-enriched *Hsd17b6* is associated with fatty liver disease. To address this question, two groups of mice were fed either the standard chow or the high-fat choline-deficient diet (HFCDA)—a diet widely applied for the model generation of fatty liver disease. As expected, the HFCDA diet challenge dramatically increased hepatic TG content (Fig. 1E) and the expression of lipid synthetic genes (*Acly*, *Fasn*, and *Dgat2*), inflammatory markers (*Il1b*, *Tnfa*, *Ccl2*, and *Tgfb*), as well as fibrotic genes (*Col1a1*, *Acta2*, and *Timp1*) (Fig. 1F), indicative of a successful model generation. Of note, the expression of *Hsd17b6* was also significantly increased in diet-induced fatty liver (Fig. 1F), suggesting that the expression of *Hsd17b6* in liver is associated with fatty liver. Taken together, our data indicates that liver-enriched *Hsd17b6* is associated with metabolic dysfunction-associated fatty liver disease.

### 3.2 Mouse model with Hsd17b6 specifically deleted in the liver is generated

**Fig. 2:**
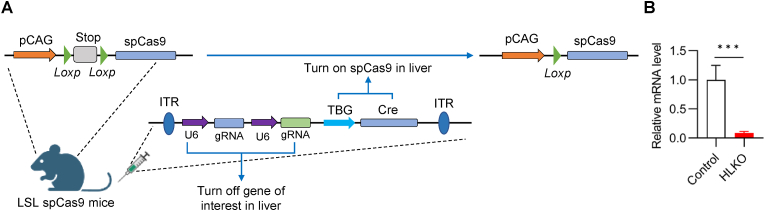
*Hsd17b6* liver-specific knockout mouse model generation. (A) The depicted view for the mouse model generation (HLKO). Briefly, two groups of 8–10 weeks male LSL-spCas9 mice were administered with AAV8 virus expressing either *Hsd17b6* or untargeted sgRNA. The virus was dosed at 5e^11^ vg/mouse. (B) Hepatic mRNA level of *Hsd17b6* tested by qPCR (the internal control, L32) (n = 4/group). Data were expressed as mean ± SEM and analyzed by Student’s t-test. ∗∗∗*p* < 0.001.

As shown in Fig. 1, our data suggests that HSD17B6 might function in the progression of fatty liver disease. However, the *Hsd17b6* knockout mouse is still lacking. To test the possibility above, we administrated adeno-associated virus serotype 8 expressing both Cre recombinase (driven by TBG, a hepatocyte-specific promoter) and *Hsd17b6* sgRNAs to Cre-dependent spCas9 knockin mice to generate a mouse model with the deletion of *Hsd17b6* in the liver (HLKO for short, hereafter) (Fig. 2A). To confirm the knockout efficiency, livers from control (administrated with the same type of virus expressing untargeted sgRNA) and HLKO mice were collected and assayed by qPCR. As shown in Fig. 2B, the mRNA level of *Hsd17b6* was dramatically downregulated in the liver of HLKO mice, reflecting an efficient deletion. The targeted region was amplified, gel-purified, and Sanger sequenced to confirm the successful edition of *Hsd17b6* (Figs. S1A, B, C, and Supplementary data 1). Knockout PCR showed a smear profile (Fig. S1B), reflecting the heterogenicity of the edited region, a feature of the CRISPR-mediated genome edition. Of note, four guide RNAs were applied in our model generation, which could further increase the heterogenicity. Taken together, the mouse model with *Hsd17b6* deletion in the liver was successfully established.

### 3.3 Hepatic *Hsd17b6* is dispensable for diet-induced fatty liver disease

**Fig. 3:**
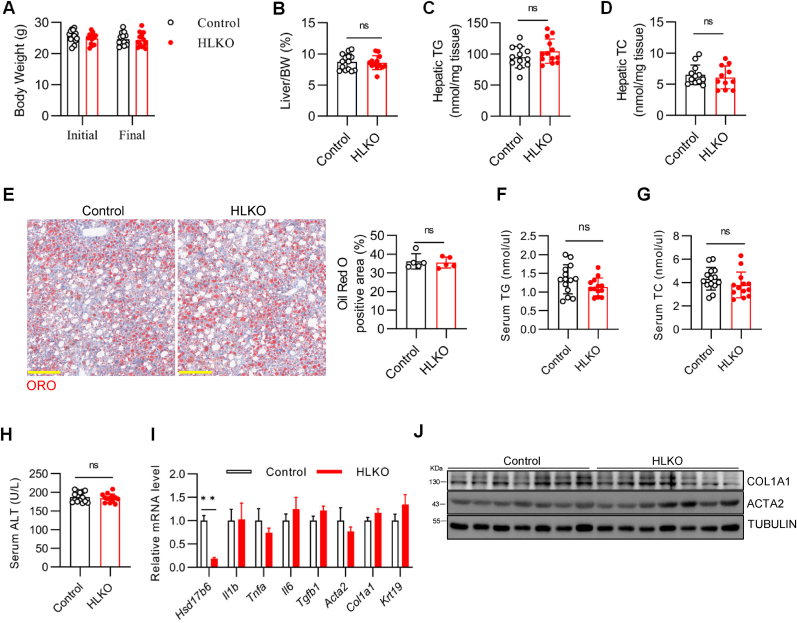
Hepatic HSD17B6 is dispensable for fatty liver induced by the high-fat choline-deficient diet (HFCDA). (A–J) Control and HLKO mice were challenged with the HFCDA diet for 8 weeks. The mice were fasted overnight and sacrificed for sampling. (A) The initial and final body weights. (B) Liver body weight ratio. (C) Liver triglyceride content. (D) Liver total cholesterol (TC). (E) Representative images of Oil red O staining of liver sections and quantification (n = 5/group) (Scale bar, 200 μm). (F) Serum triglyceride content. (G) Serum total cholesterol. (H) Serum alanine aminotransferase (ALT) activity. (I) Hepatic expression of inflammatory markers (*Il1b*, *Tnfa*, *Il6*, and *Tgfb1*), and fibrotic markers (*Acta2*, *Col1a1*, and *Krt19*) assayed by qPCR (n = 12/group). L32 was used as an internal control. (J) Western blotting analysis by using the antibodies as indicated. n = 14 or 13/group for panels A, B, C, D, F, G, and H. Data were expressed as mean ± SEM and analyzed by Student’s t-test. ∗∗*p* < 0.01, ns denotes not significant (*p* > 0.05).

To test the role of *Hsd17b6* in fatty liver disease, control, and HLKO mice were challenged with the HFCDA diet for 8 weeks, an experimental setting widely applied [[11], [12], [13]]. Both initial and final body weights were comparable between the two genotypes (Fig. 3A). So were the liver body weight ratios (Fig. 3B). Of note, it is reported that the liver body weight of mice on chow is around 4 % [7,8], here, the liver body weight ratios were around 8 %, indicating a phenotype of hepatomegaly—a typic feature of fatty liver disease. Next, the hepatic triglyceride and total cholesterol were measured and turned out to be comparable between the two genotypes (Fig. 3C and D). In line with the finding above, Oil red O staining suggests that the levels of hepatic fat accumulation were comparable between the two genotypes (Fig. 3E).

Next, we measured serum triglyceride and total cholesterol and found that they were not altered by the hepatic deletion of *Hsd17b6* (Fig. 3F and G). The levels of alanine transaminase (ALT), one typic marker of liver injury, were the same between control and HLKO mice (Fig. 3H). Lipotoxicity-induced liver injury contributes to the development of inflammation and fibrosis [14]. Therefore, the inflammation markers were tested by qPCR assay, and found that they were comparable between the two genotypes (Fig. 3I). The expression of fibrotic markers including *Acta2*, *Col1a1*, and *Krt19* was not affected by the hepatic deletion of *Hsd17b6* either (Fig. 3I). Immunoblotting using antibodies against COL1A1, and ACTA2 indicates that the levels of fibrosis were also comparable between the two groups (Fig. 3J). Taken together, our data suggests that liver *Hsd17b6* is dispensable for HFCDA-induced fatty liver disease.

Based on the tissue distribution profile (BioGPS), fourteen genes encoding members of *17β-HSDs* family of mice could be classified into 4 types: 1) specifically and highly expressed in liver, similar to *Hsd17b6* (Fig. 2SA) (*Hsd17b2*, *Hsd17b5*, and *Hsd17b13*) (Fig. 2SB, C, and D); 2) ubiquitously expressed in most of the tissues and highly expressed in liver (*Hsd17b4* and *Hsd17b8*) (Fig. 2SE and F); 3) ubiquitously expressed in most of the tissues and moderately expressed in liver (*Hsd17b7*, *Hsd17b10*, *Hsd17b11*, and *Hsd17b12*) (Fig. 2SG, H, I, and J); 4) lowly expressed in liver (*Hsd17b1*, *Hsd17b3*, *Hsd17b9*, and *Hsd17b14*) (Fig. 2SK, L, M and N). Accordingly, ten of 17β-HSDs, but not HSD17B1, HSD17B3, HSD17B9, and HSD17B14, were detected by our proteomic analysis, and their protein levels were not affected by hepatic deletion of *Hsd17b6* (Fig. S3A). Interestingly, hepatic deletion of *Hsd17b6* only slightly decreases the level of hepatic estrone (*p* = 0.33) (Fig. S3B). Of note, other 17β-HSDs, such as HSD17B4, could generate estrone as well [15]. In addition, the protein level of HSD17B6 is much lower than some of the 17β-HSDs, such as HSD17B4, HSD17B5, and HSD17B10 (Fig. S3C), indicating that the role of HSD17B6 in steroid synthesis might be minimal in the liver. Taken together, the estrone synthetic activity of liver with *Hsd17b6* deletion might be compensated by other 17β-HSDs such as HSD17B4, likely, which partially explains the lack of phenotype.

### 3.4 Hsd17b6 knockout liver presents a special proteome signature

**Fig. 4:**
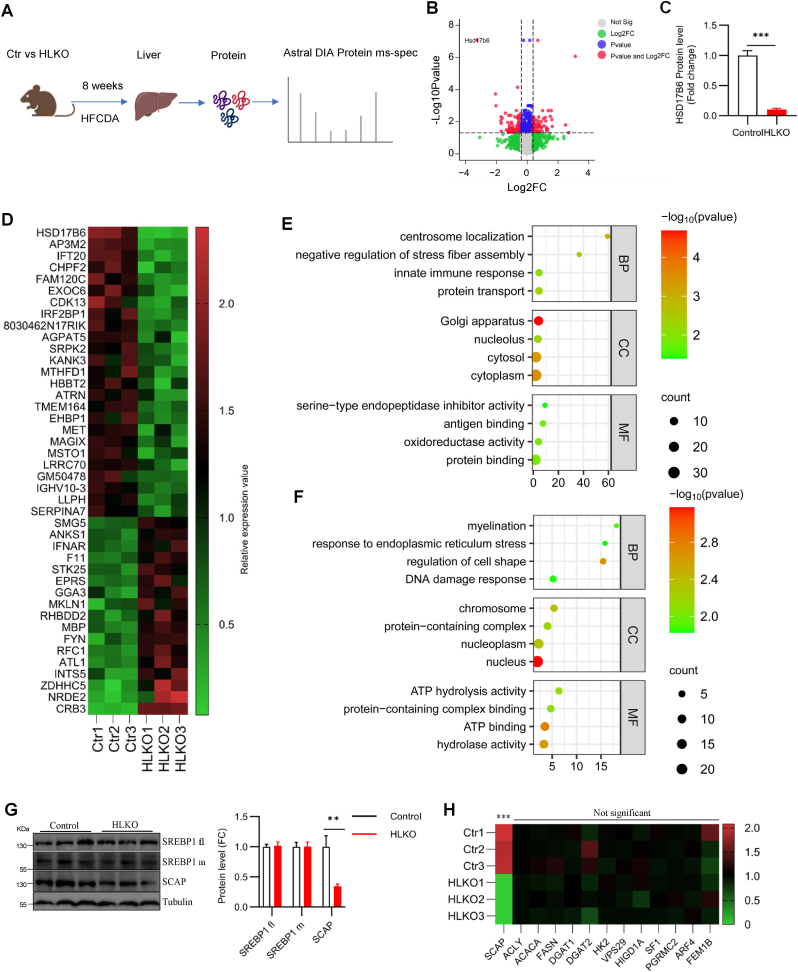
The *Hsd17b6* deleted liver presents a special proteome signature. (A–F) Control and HLKO mice were subjected to the high-fat choline-deficient diet (HFCDA) challenge for 8 weeks. The mice were fasted overnight and sacrificed for liver proteomic analysis (n = 3/group). (A) The workflow for Astral DIA-based protein mass-spec analysis. (B) The volcano plot presents the protein expression profile. (C) Histogram plotting the HSD17B6 protein level from proteomic data (n = 3). (D) The heatmap presents the altered hits (Fold change >2.0 and *p* < 0.05). BP, biological process; MF, molecular function; CC, cellular component. (E) Downregulated and (F) upregulated proteins (Fold change >2.0 and *p* < 0.05) are subjected to GO enrichment analysis and the top 4 from each category are presented in a bubble plot (Bonferroni FDR <0.05). (G) WB for liver lysate against antibodies as indicated (n = 6). (H) The protein expression level of SREBP regulator SCAP and other targets of SREBP mined from proteomic data are presented in the heatmap. Data were expressed as mean ± SEM and analyzed by Student’s t-test. ∗∗∗*p* < 0.001.

Since our data suggests that hepatic deletion of *Hsd17b6* does not affect diet-induced fatty liver (Fig. 3), we’d like to further confirm the loss of *Hsd17b6* at the protein level, and thoroughly assess the effect of hepatic *Hsd17b6* on the proteome. To this end, livers from control and HLKO mice were subjected to proteomic analysis (Fig. 4A). There were 78 upregulated hits and 59 downregulated hits (fold change (FC) > 1.3, *p* < 0.05) (Fig. 4B). Of note, HSD17B6 protein level was dramatically decreased with *Hsd17b6* deletion (Fig. 4C), verifying the hepatic loss of *Hsd17b6* at the protein level. Different expressed proteins (DEPs) (FC > 2.0 and *p* < 0.05) were presented in the heatmap (Fig. 4D). The top three upregulated proteins are CRB3, NRDE2, and ZDHHC5 (Fig. 4D). Interestingly, the top three downregulated proteins, AP3M2, IFT20, and CHPF2, seem to be located in the Golgi apparatus [[16], [17], [18]].

Gene ontology annotation was performed for DEPs based on three categories: biological process (BP), molecular function (MF), and cellular component (CC). The top four of each category from downregulated proteins were presented in Fig. 4E. Of the BP terms, DEPs are enriched in centrosome localization, negative regulation of stress fiber assembly, innate immune response, and protein transport (Fig. 4E). Of the CC terms, DEPs are enriched in the Golgi apparatus, nucleolus, cytosol, and cytoplasm (Fig. 4E). Of the MF terms, DEPs are enriched in serine-type endopeptidase inhibitor activity, antigen binding, oxidoreductase activity, and protein binding (Fig. 4E). The top four of each category from upregulated proteins were presented in Fig. 4F. Of the BP terms, DEPs are enriched in myelination, response to endoplasmic reticulum stress, regulation of cell shape, and DNA damage response (Fig. 4F). Of the CC terms, DEPs are enriched in chromosome, protein-containing complex, nucleoplasm, and nucleus (Fig. 4F). Of the MF terms, DEPs are enriched in ATP hydrolysis activity, protein-containing complex binding, ATP binding, and hydrolase activity (Fig. 4F). The specific proteins from up and downregulated pathways were presented in Table S2 and Table S3, respectively. Taken together, HLKO liver shows a special proteome signature, likely, with DEPs enriched in Golgi apparatus.

SREBPs, including SREBP1a, SREBP1c, and SREBP2, are a family of transcription factors that transactivate essential genes of *de novo* lipid synthesis [19]. Interestingly, Wei et al. found that HSD17B6 binds to the SREBPs and inhibits SREBP signaling independent of its steroid synthesis activity [4]. Furthermore, they documented that *Hsd17b6* overexpression inhibits the expression of SREBPs and their target genes [4]. Following this logic, *Hsd17b6* deletion should promote the expression of SREBPs and their target genes. However, both protein and mRNA levels of SREBP1 were not affected (Fig. 4G, Fig. S3D), as well as its target genes, including *Fasn*, *Acly*, and *Dgat* etc (Fig. 4H, and Fig. S3D). SREBP cleavage activating protein (SCAP), a key regulator of SREBP maturation, induces translocation of SREBP from the endoplasmic reticulum to the Golgi apparatus, allowing its maturation [20]. Interestingly, in our experiment setting, *Hsd17b6* deletion significantly downregulates the protein level of SCAP (Fig. 4G and H), which likely counteracts the promotion of SREBP activity induced by *Hsd17b6* deletion and ultimately leads to no significant impact on mouse phenotype. Of note, the mRNA level of *SCAP* was not affected by *Hsd17b6* deletion (Fig. S3D), reflecting a posttranscriptional regulation.

## 4 Conclusion

HSD17B6, encoded by *Hsd17b6*, is an enzyme involved in the synthesis of steroids, including estrone. Here, we found that *Hsd17b6* is enriched in the liver of both humans and mice. The expression of *Hsd17b6* is correlated with diet-induced fatty liver disease, indicating it might play a role in the progression of MAFLD. However, our data shows that the deletion of *Hsd17b6* in the liver does not affect high-fat choline-deficient diet-induced fat accumulation, inflammation, or fibrosis.

This study was conducted in male mice, and females should be included in the future, considering the role of HSD17B6 in the synthesis of steroids—a type of sex hormone. The hepatic level of estrone is not affected by hepatic deletion of *Hsd17b6*, which might be one of the reasons behind the lack of metabolic phenotype. Relatively, the hepatic protein level of HSD17B6 is low, compared to some members of the 17β-HSDs family, indicating that the role of HSD17B6 in estrone synthesis might be minimal and negligible. Of course, measurements of estrone and androsterone, especially in circulation, should be considered in future studies as well.

Interestingly, HSD17B6 appears to function independently of its steroid synthesis activity [4]. Our data suggests that HSD17B6 regulates SCAP post-transcriptionally. However, the link is still missing between this regulatory role and metabolic phenotype. To establish this link, the relationship between HSD17B6 and SCAP needs to be further confirmed and illustrated. In addition, different experimental settings, such as dietary selection, should be considered as well.

## CRediT authorship contribution statement

**Delong Yuan:** Investigation. **Nan Bai:** Investigation. **Qihan Zhu:** Investigation. **Shaoxuan Song:** Investigation. **Anyuan He:** Writing – review & editing, Funding acquisition, Conceptualization. **Jianqing Wang:** Writing – review & editing, Funding acquisition, Conceptualization. **Yali Chen:** Writing – review & editing, Writing – original draft, Investigation, Funding acquisition.

## Ethics statement

All animal experiments were approved and performed following the guidelines of the Animal Ethics Committees at Anhui Medical University (Permission No. LLSC20210007. March 1, 2021).

## Funding

This work was supported by 10.13039/501100001809National Natural Science Foundation of China (32370738 to A.H.). Fund for Colleges and Universities of Anhui Province (KJ2021A0243 to Y.C.).

## Declaration of competing interest

We declare that this manuscript is original, has not been published before, and is not currently being considered for publication elsewhere. All authors have reviewed this manuscript and approved this submission.

## Data Availability

All necessary experimental raw data were uploaded to Figshare via link below: https://figshare.com/s/bbbe246dacf6304786ef
