# Supplementary material for: Hepatic HSD17B6 is dispensable for diet-induced fatty liver disease in mice
Source: Biochem Biophys Rep. 2025 Jan 19;41:101924. doi: 10.1016/j.bbrep.2025.101924 (PMC11787692; doi:10.1016/j.bbrep.2025.101924)
Supplement: Multimedia component 1 [file mmc1.pdf]

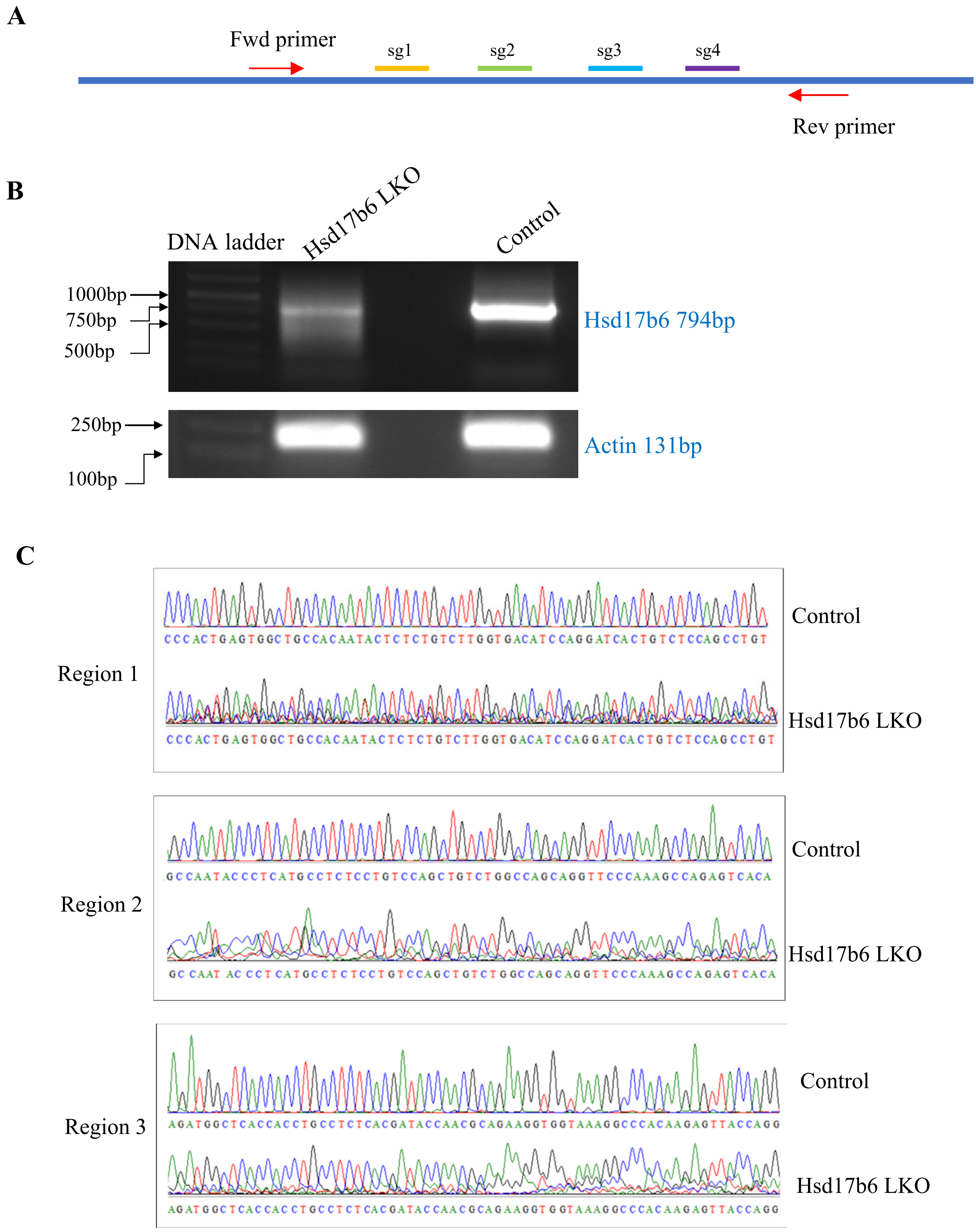

Fig S1. Hsd17b6 KO validation by PCR and Sanger sequencing. (A) Schematic view of sites of guide RNAs and primers for the amplification of targeted region. The Fwd and Rev primer sequences are: *aag acc tgg act tgg act* and *gtc ttg aag ctcc cag gt*. (B) Hepatic cDNAs were used as templates to amplify the targeted region using Fwd and Rev primer and analyzed by gel. (C) The major bands of panel B were gel-purified and subjected to Sanger sequencing. Some representative region were presented. The full sequences were provided in **Supplement data 1**.
