# Supplementary material for: Hepatic HSD17B6 is dispensable for diet-induced fatty liver disease in mice
Source: Biochem Biophys Rep. 2025 Jan 19;41:101924. doi: 10.1016/j.bbrep.2025.101924 (PMC11787692; doi:10.1016/j.bbrep.2025.101924)

A

Hsd17b6

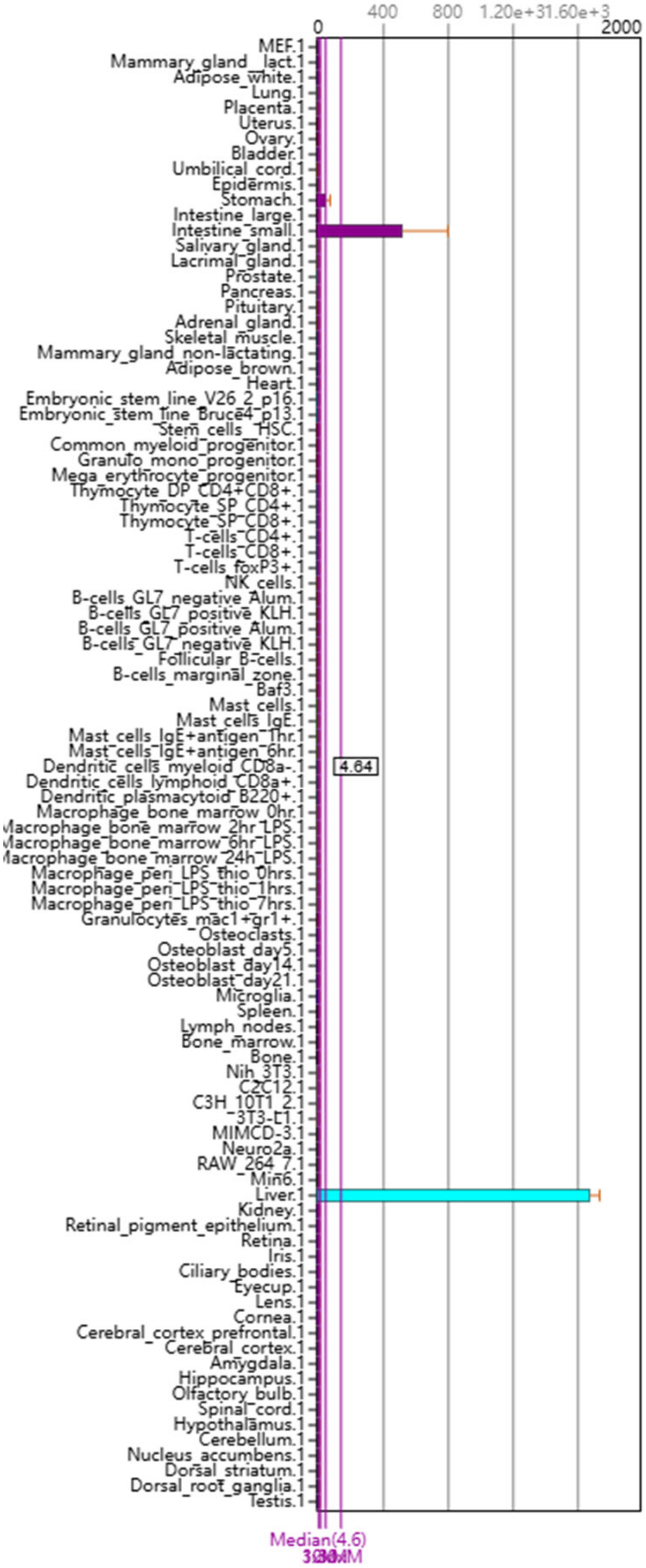

B

Hsd17b5

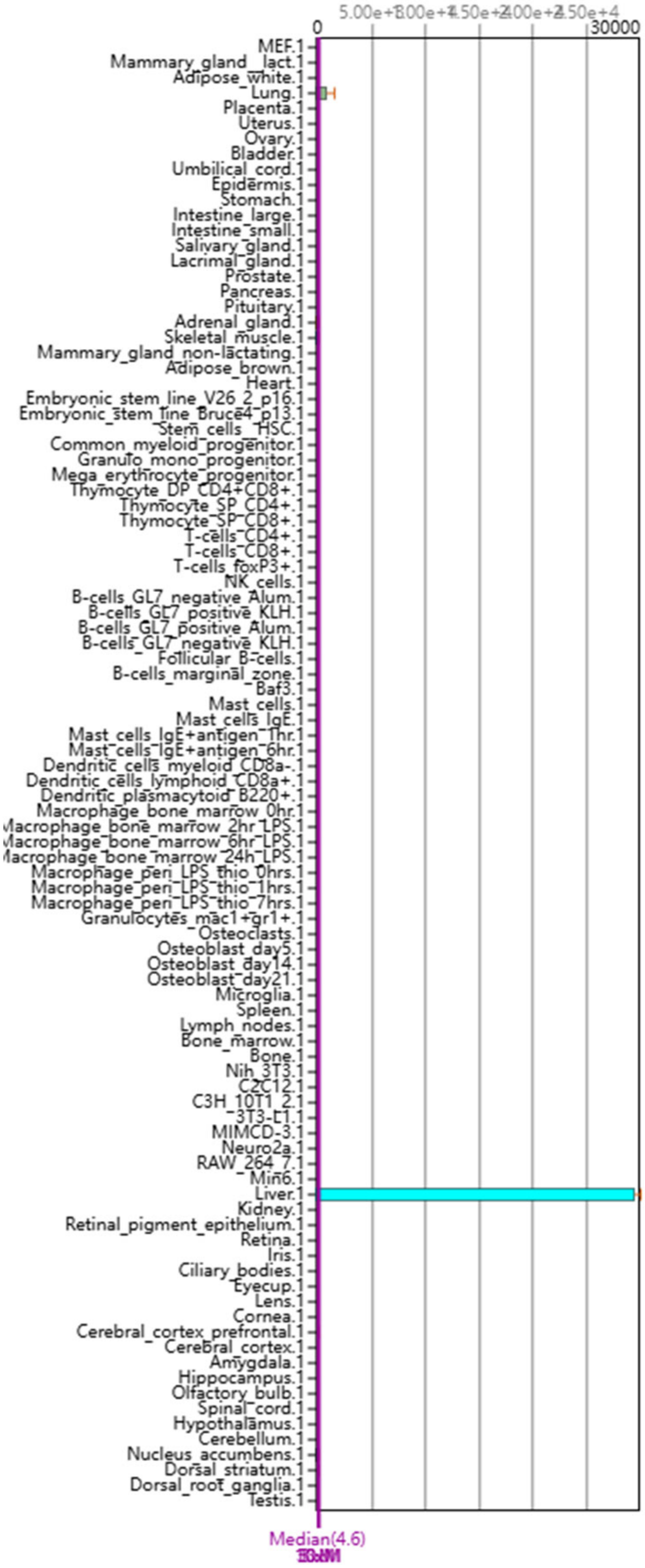

Fig S2. Expression profiles of mouse genes encoding 17β-HSDs family. Data was mined from BioGPS. (A) *Hsd17b6*; (B) *Hsd17b5*; (C) *Hsd17b2*; (D) *Hsd17b13*; (E) *Hsd17b4*; (F) *Hsd17b8*; (G) *Hsd17b7*; (H) *Hsd17b10*; (I) *Hsd17b11*; (J) *Hsd17b12*; (K) *Hsd17b1*; (L) *Hsd17b3*; (M) *Hsd17b9*; (N) *Hsd17b14*.

Fig S2. Continued.

C

Hsd17b2

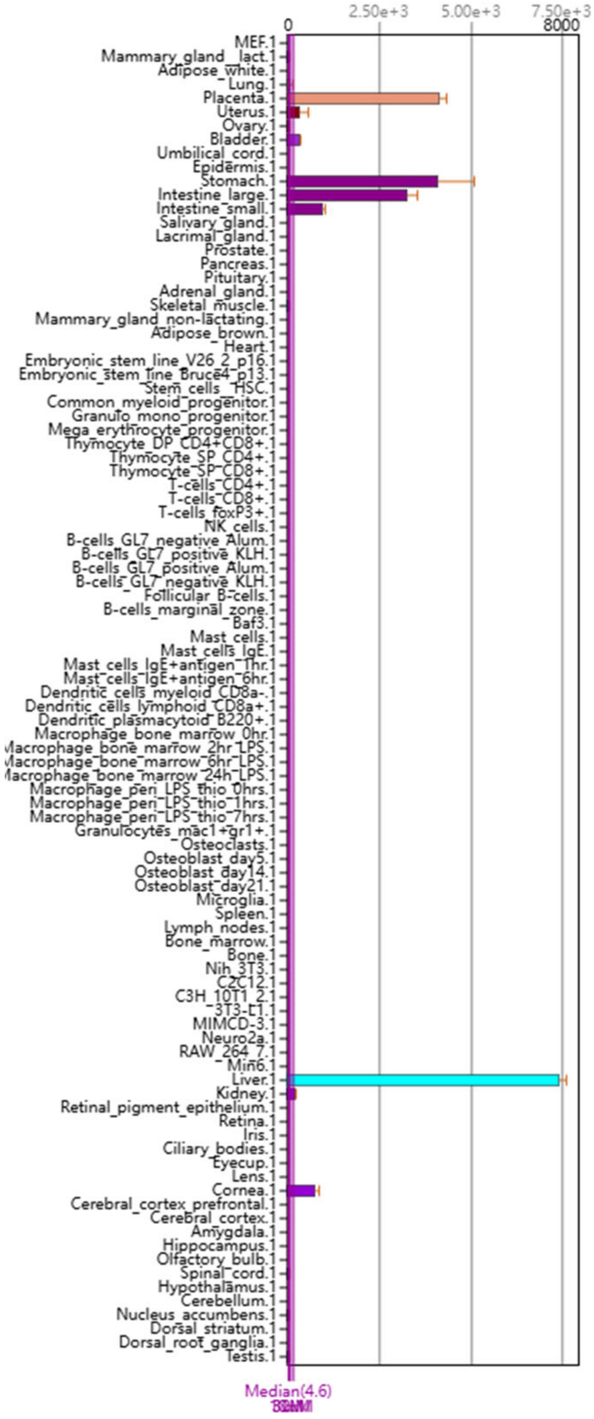

D

Hsd17b13

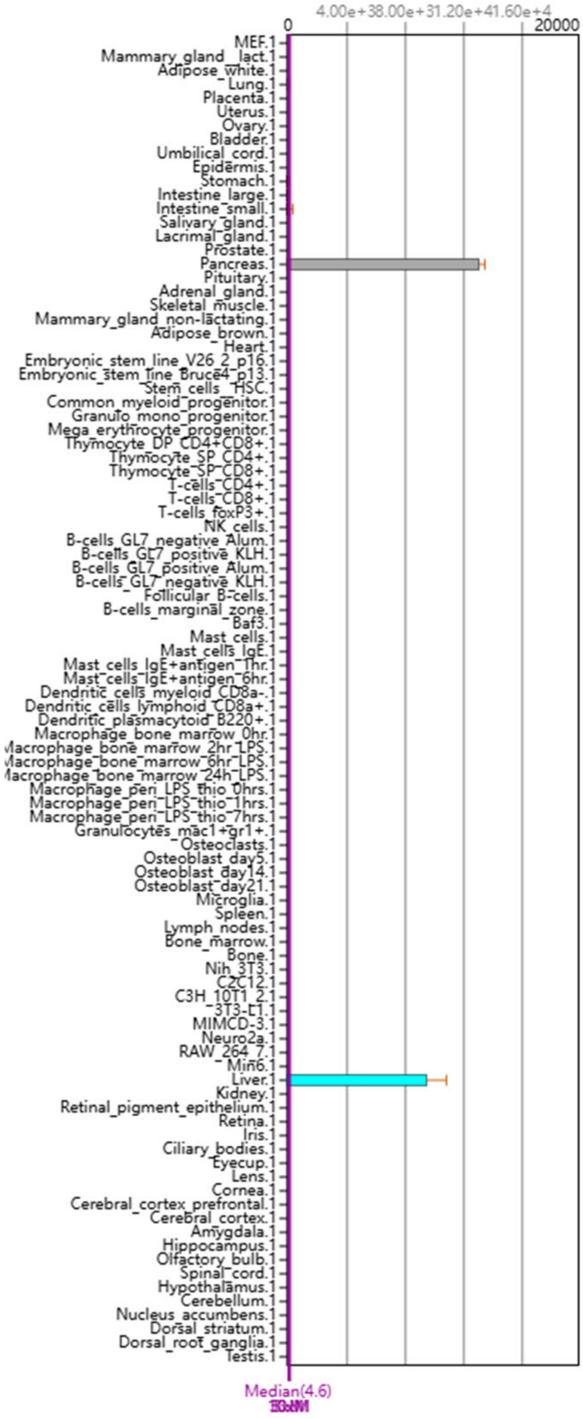

Fig S2. Continued.

E

Hsd17b4

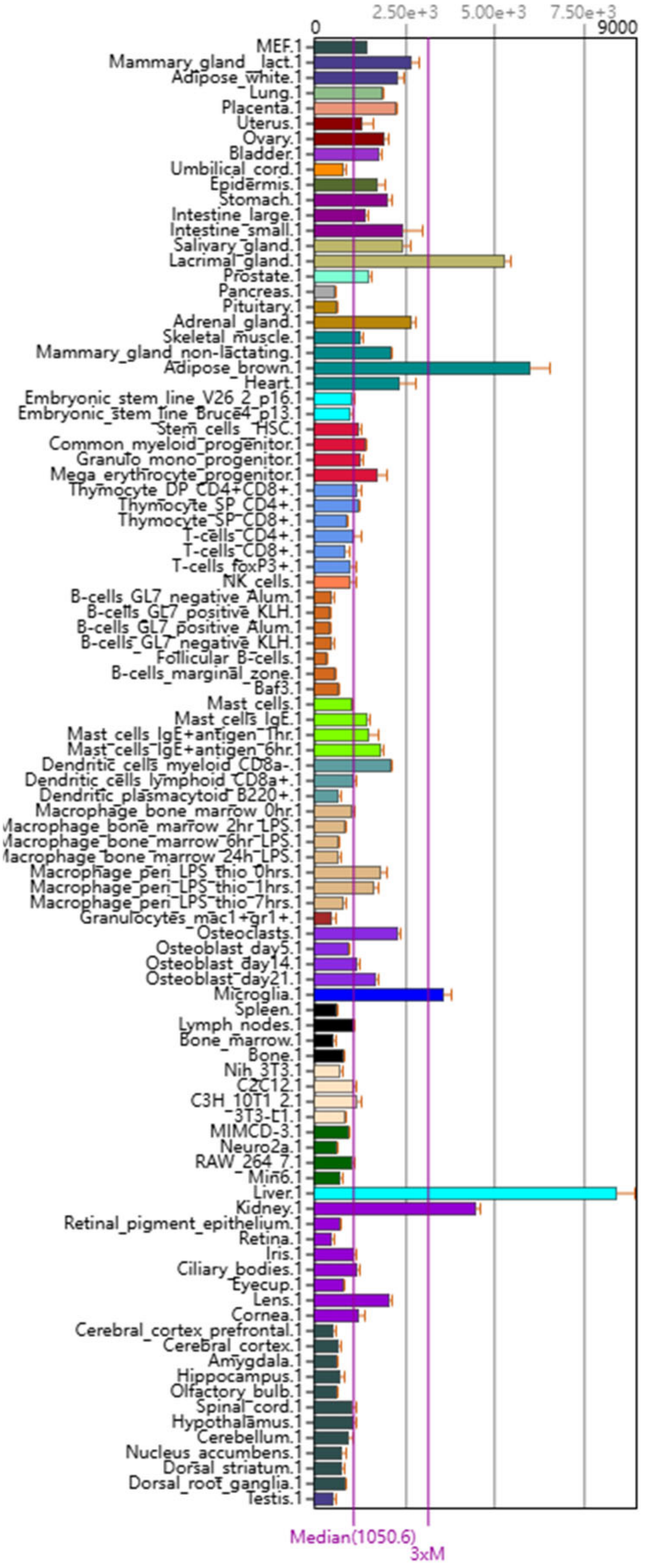

F

Hsd17b8

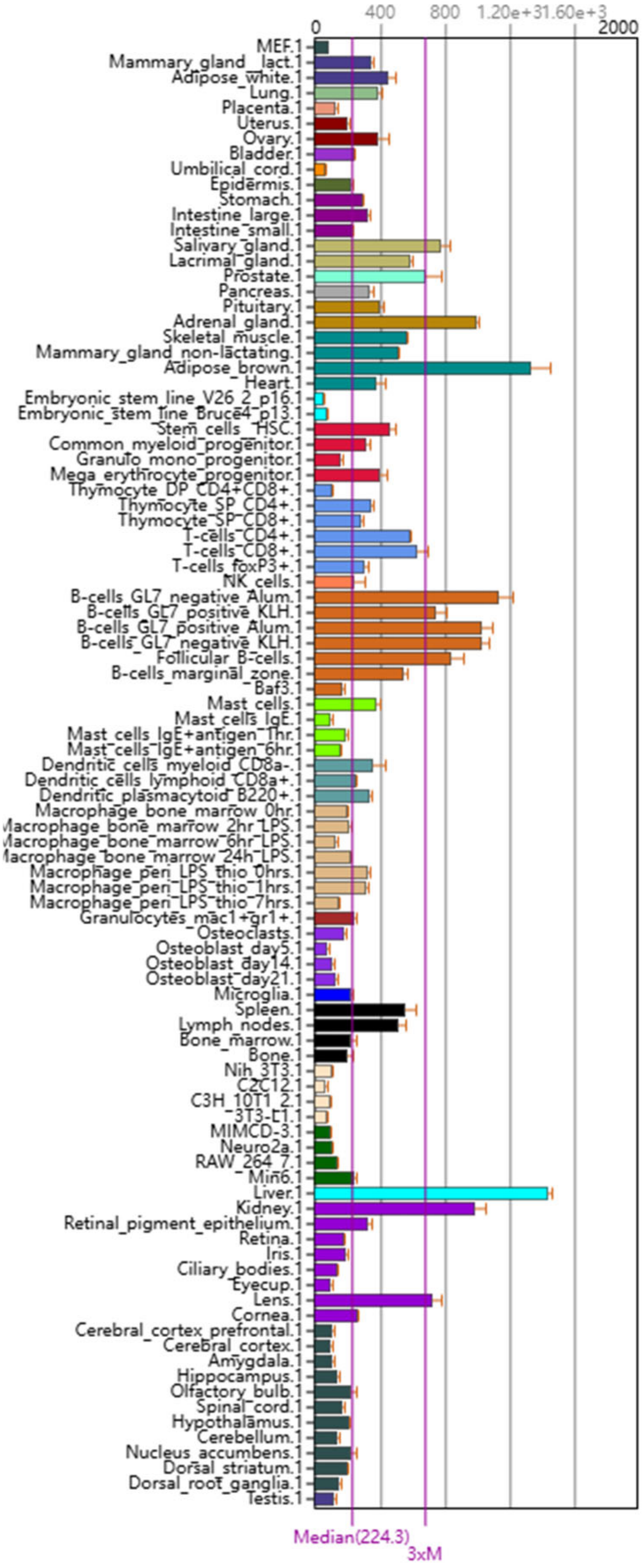

Fig S2. Continued.

G

Hsd17b7

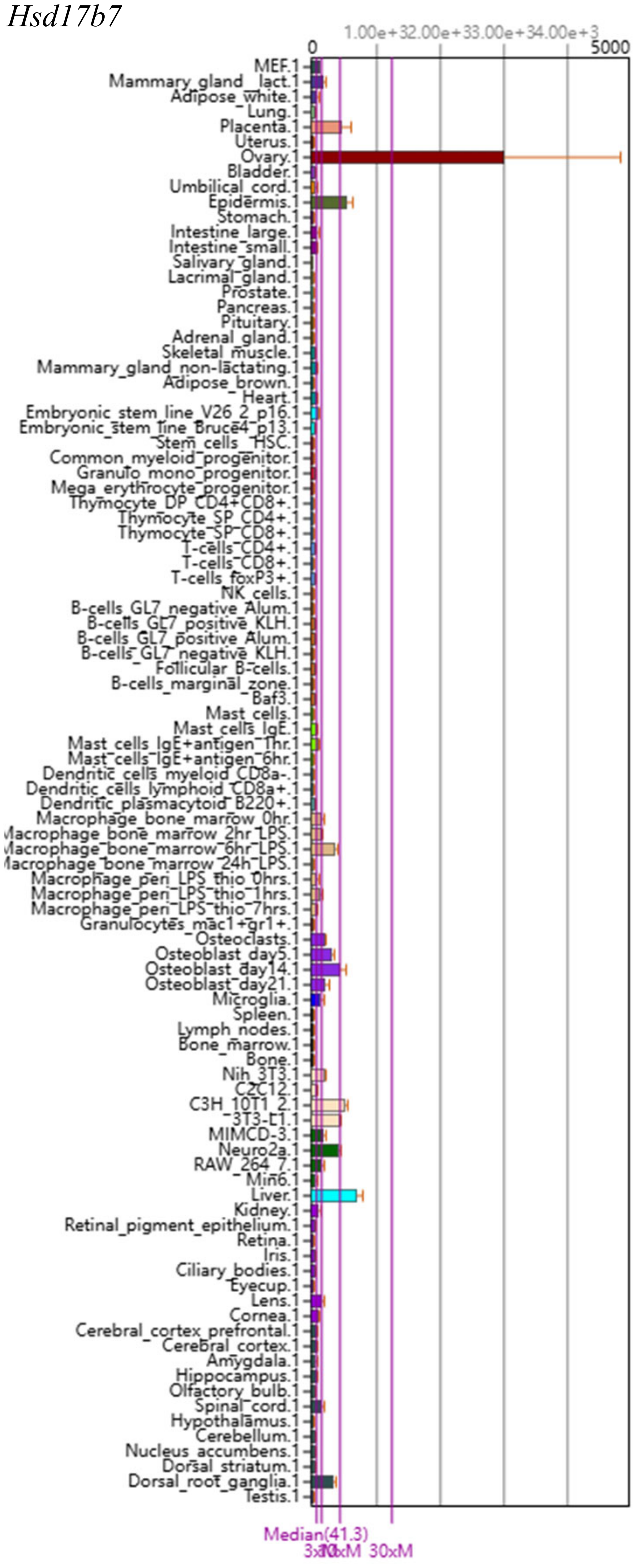

H

Hsd17b10

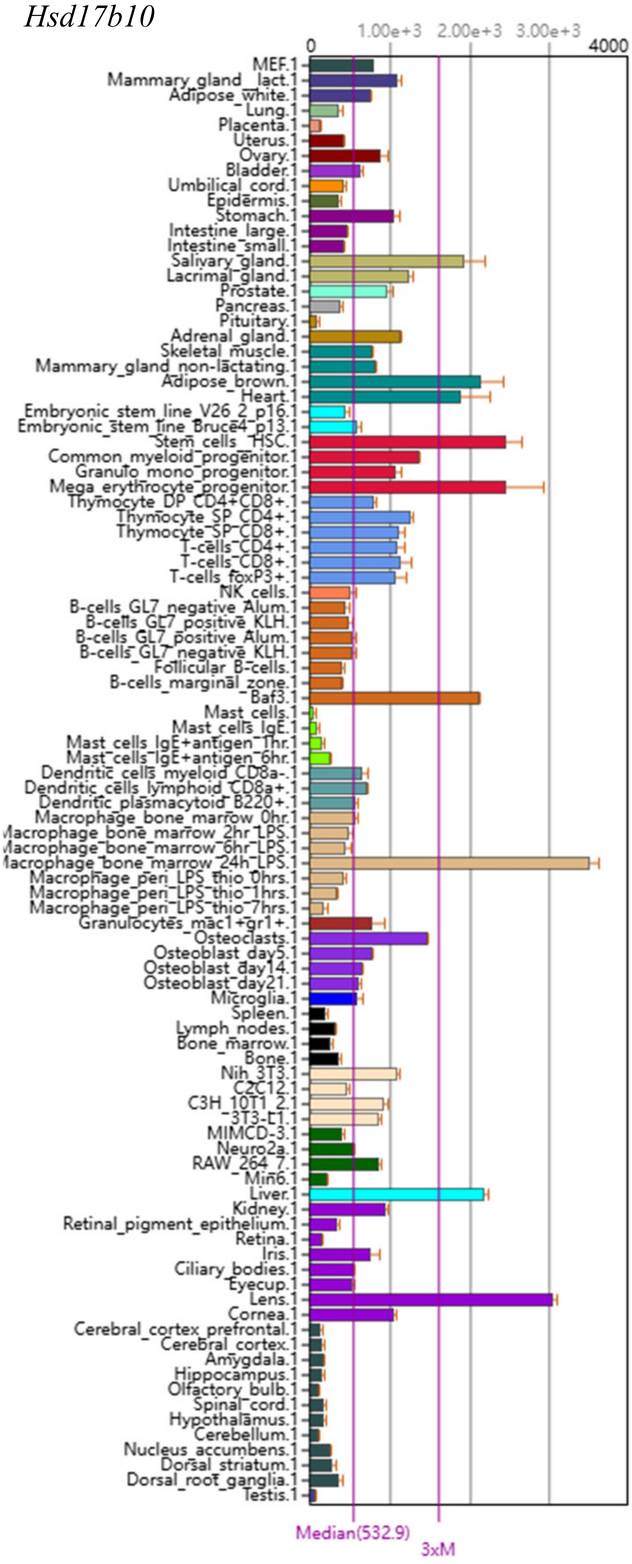

Fig S2. Continued.

I

Hsd17b11

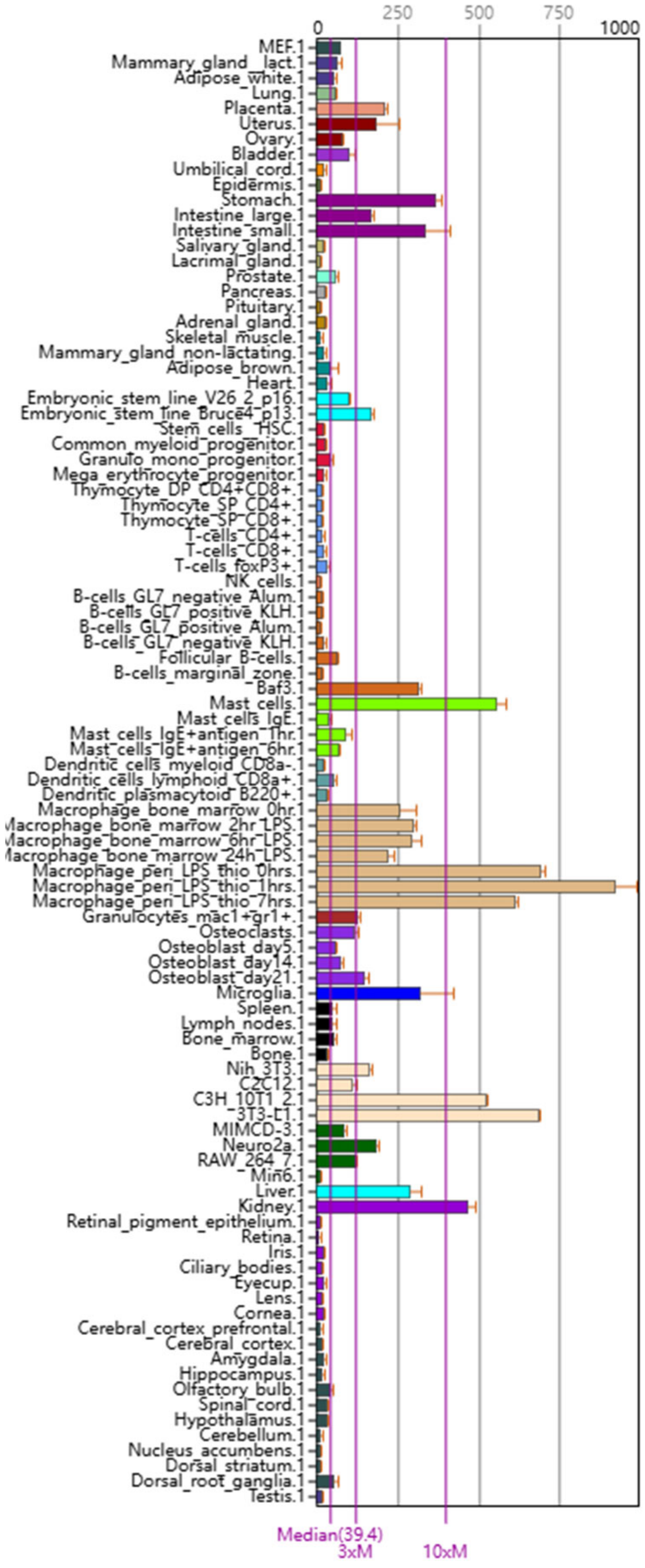

J

Hsd17b12

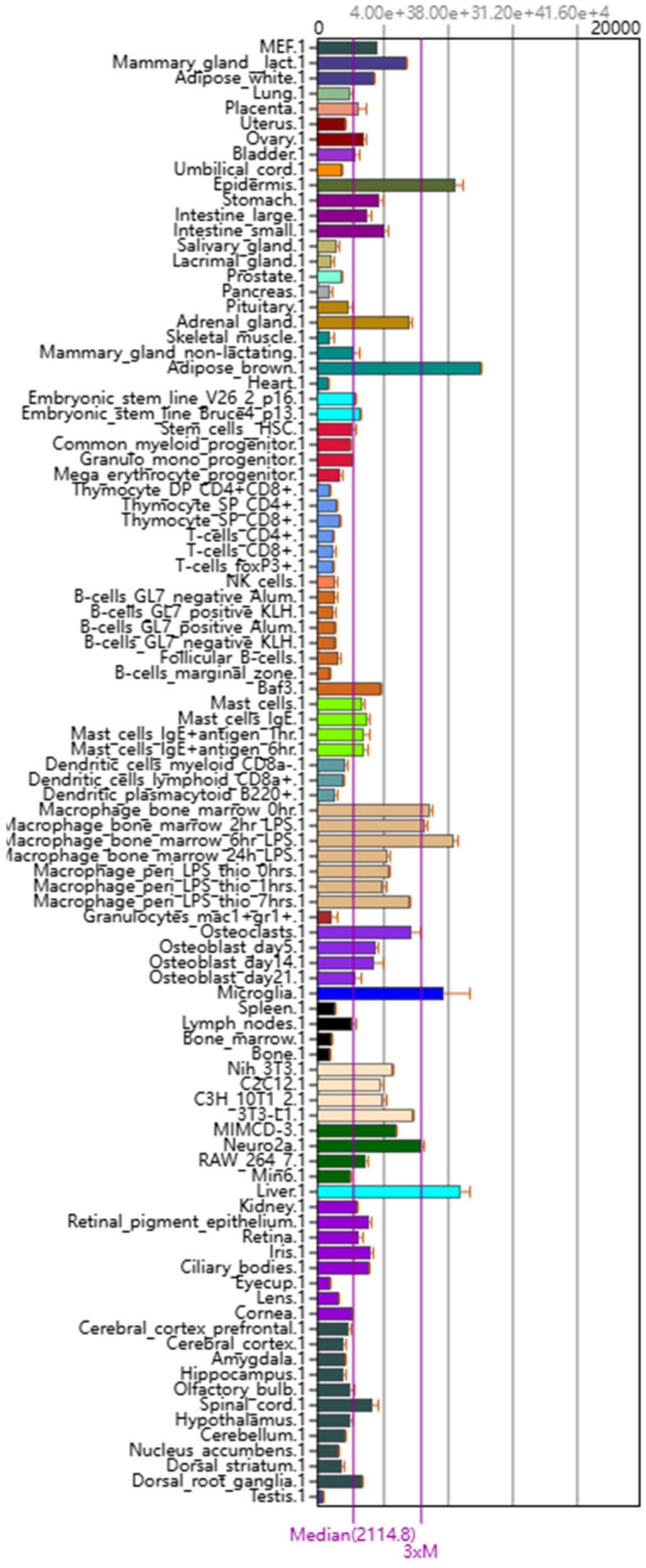

Fig S2. Continued.

K

Hsd17b1

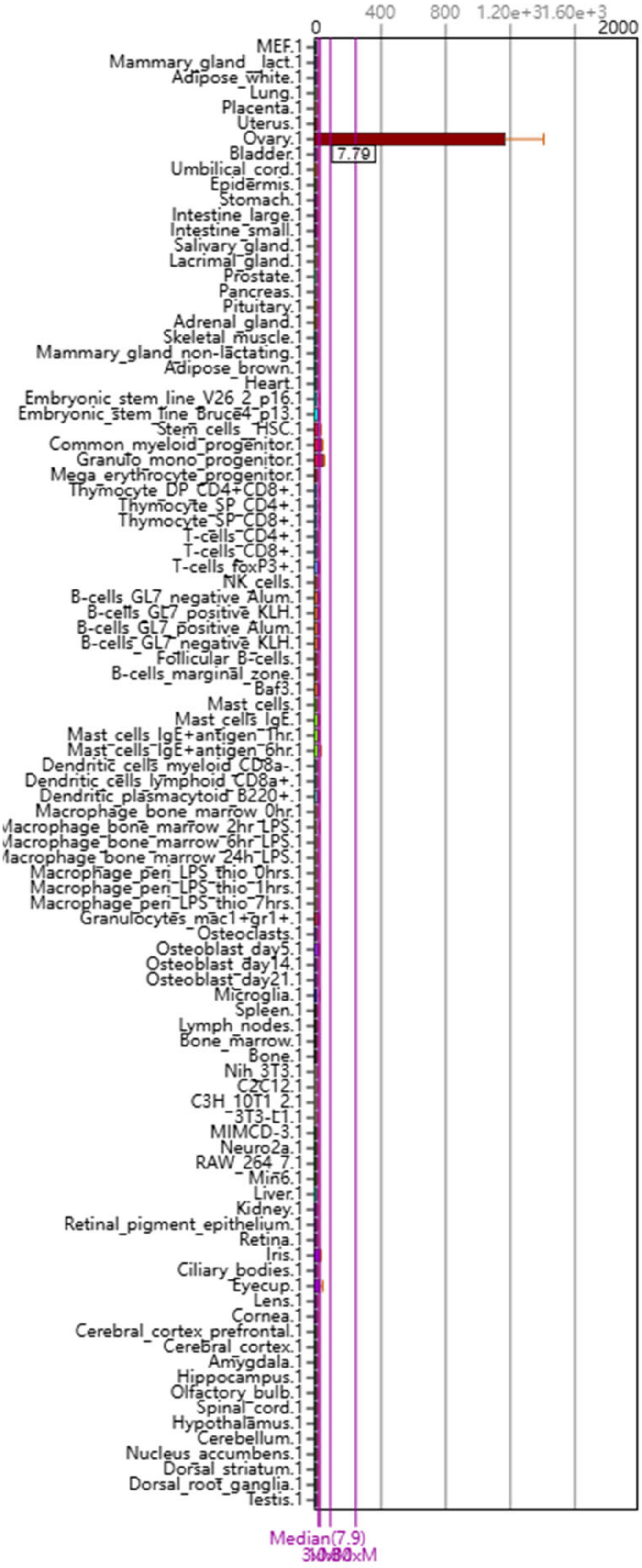

L

Hsd17b3

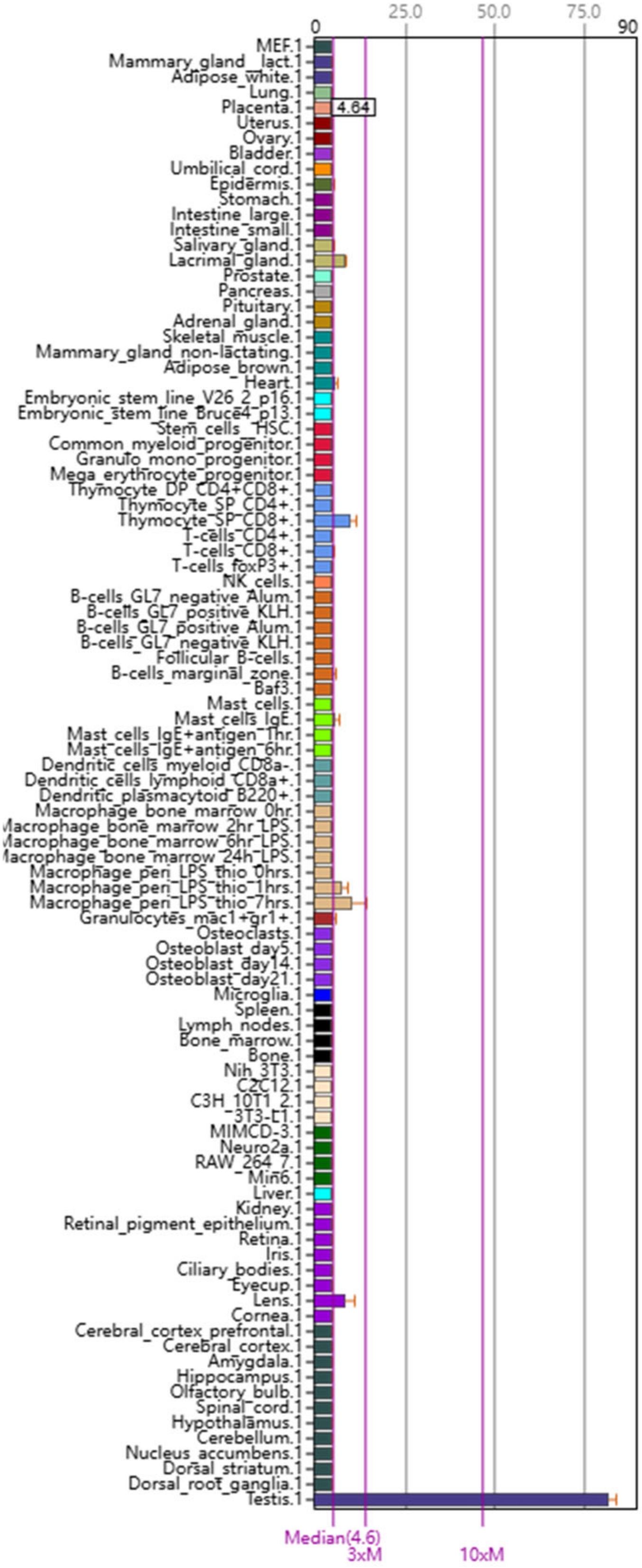

Fig S2. Continued.

M

Hsd17b9

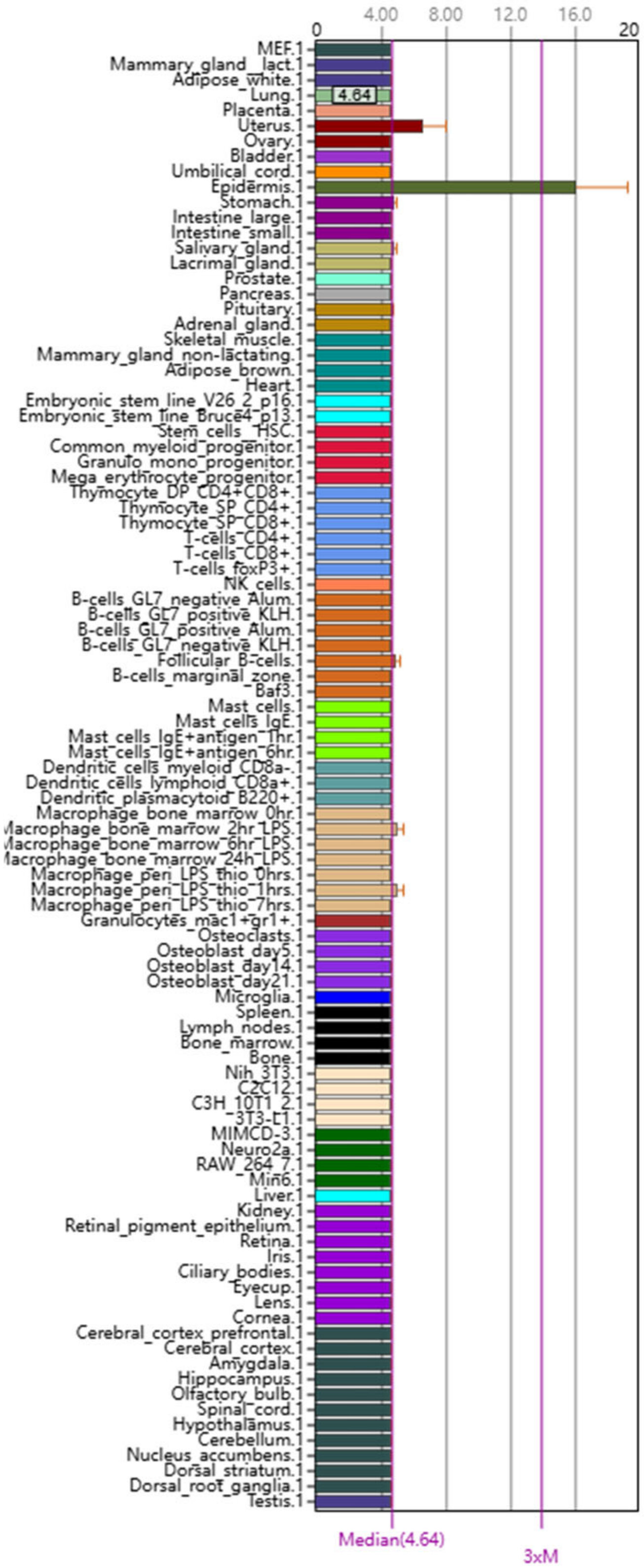

N

Hsd17b14

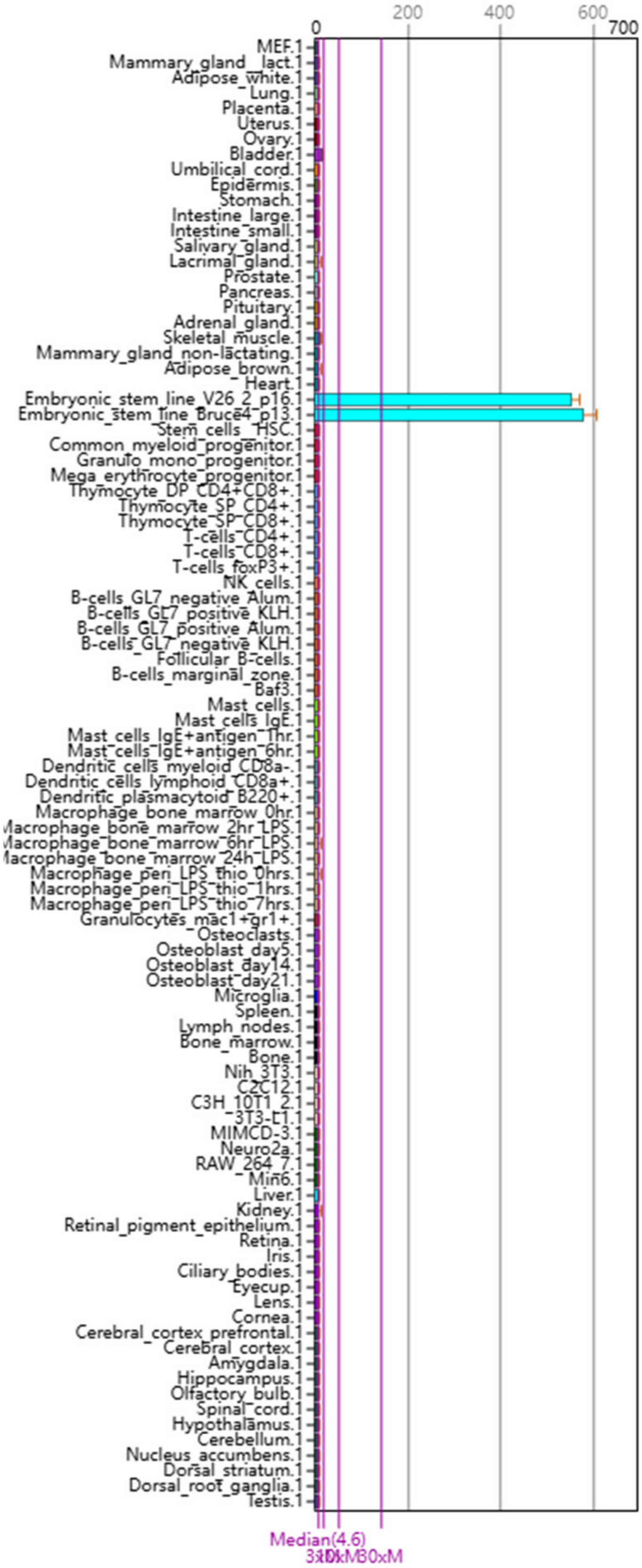

Supplement: Multimedia component 2 [file mmc2.pdf]
