# Supplementary material for: Hepatic HSD17B6 is dispensable for diet-induced fatty liver disease in mice
Source: Biochem Biophys Rep. 2025 Jan 19;41:101924. doi: 10.1016/j.bbrep.2025.101924 (PMC11787692; doi:10.1016/j.bbrep.2025.101924)
Supplement: Multimedia component 3 [file mmc3.pdf]

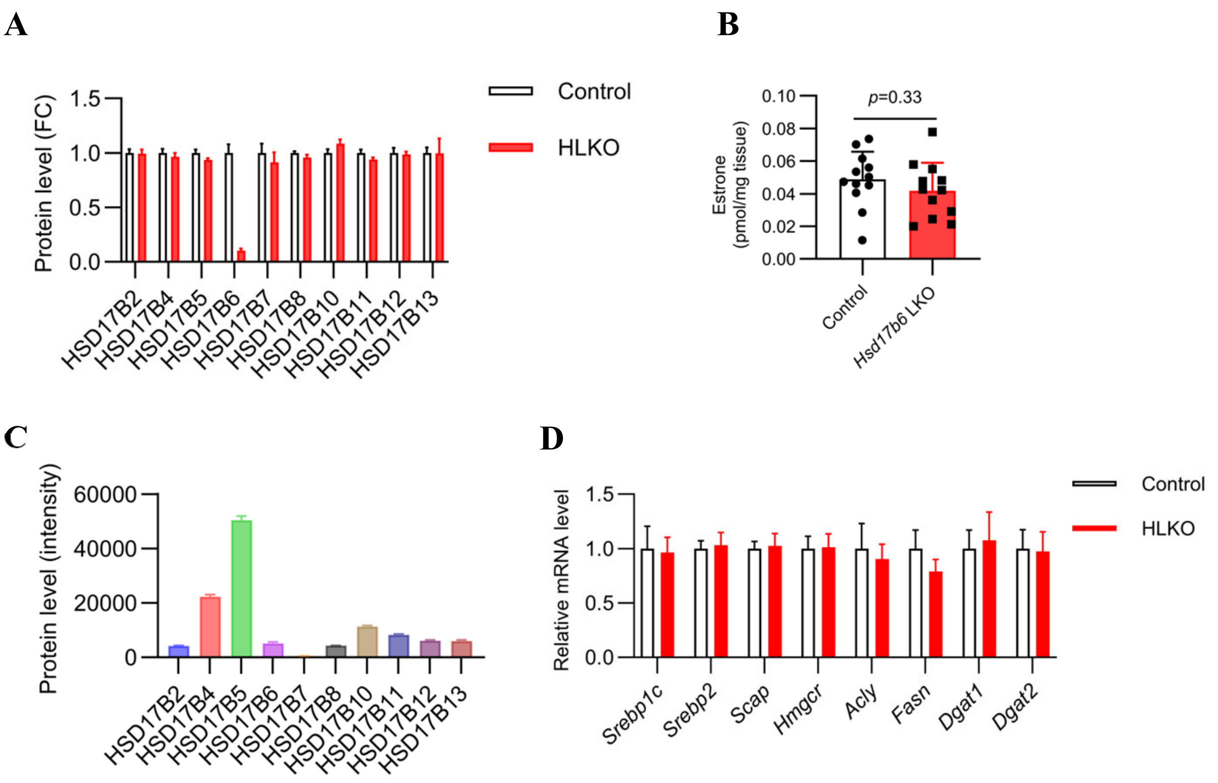

Fig S3. Related to Fig 4. (A) relative protein level of 17β-HSDs identified by proteomics (n = 3/group). (B) The level of hepatic estrone (n = 12/group). (C) Protein levels of 17β-HSDs identified by proteomics in control liver (D). Relative mRNA levels of Srebps and their target genes (n = 12/group). Data were expressed as mean ± SEM and analyzed by Student's t-test. **p* < 0.05, ***p* < 0.01, ****p* < 0.001.
