# Supplementary figures and images for: Hepatic HSD17B6 is dispensable for diet-induced fatty liver disease in mice
Source: Biochem Biophys Rep. 2025 Jan 19;41:101924. doi: 10.1016/j.bbrep.2025.101924 (PMC11787692; doi:10.1016/j.bbrep.2025.101924)

Fig 3J

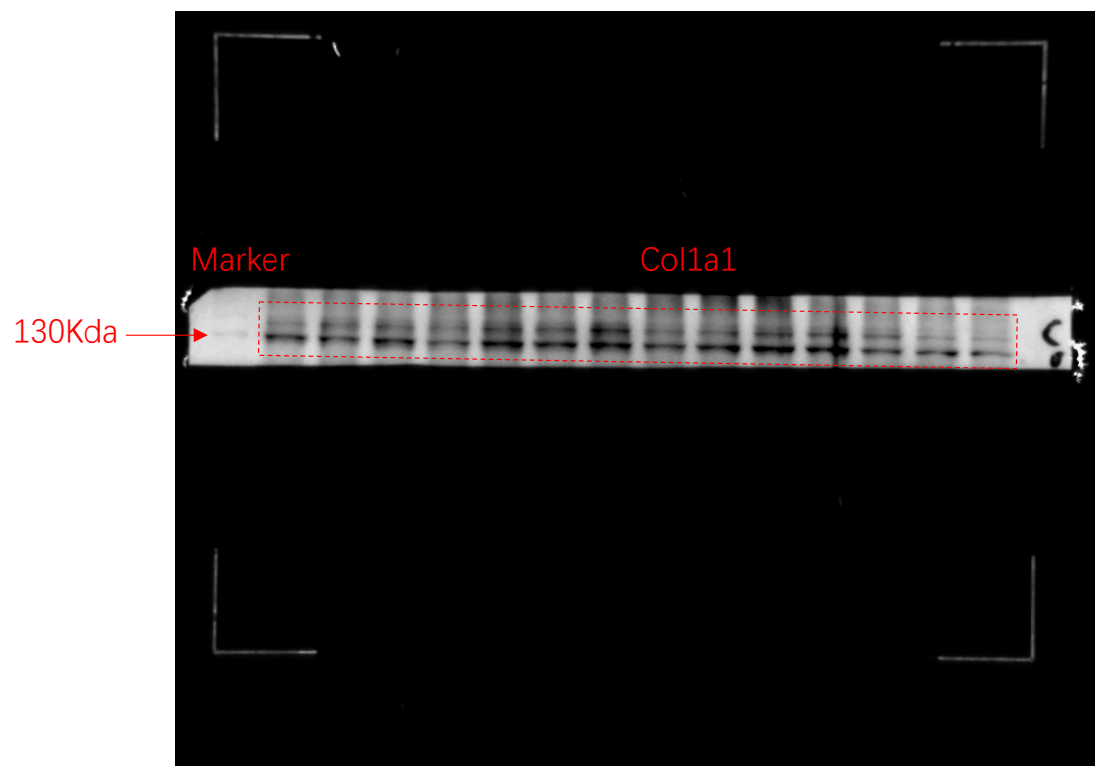

Fig 3J

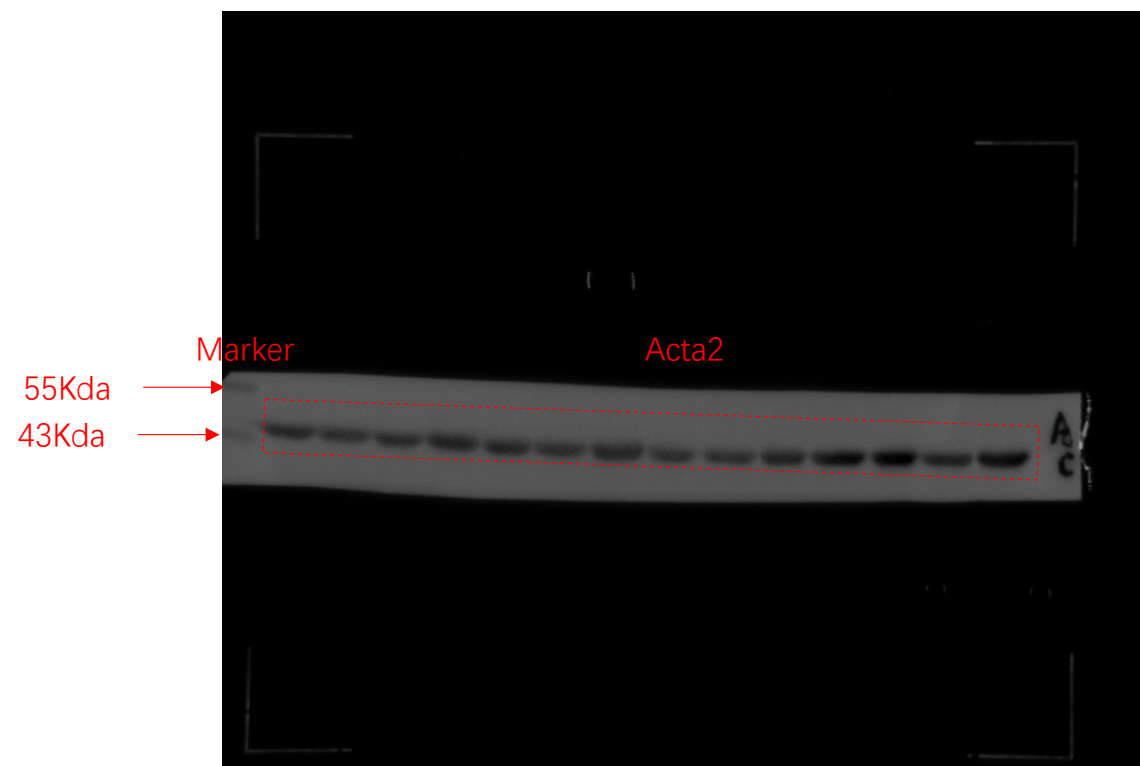

Fig 3J

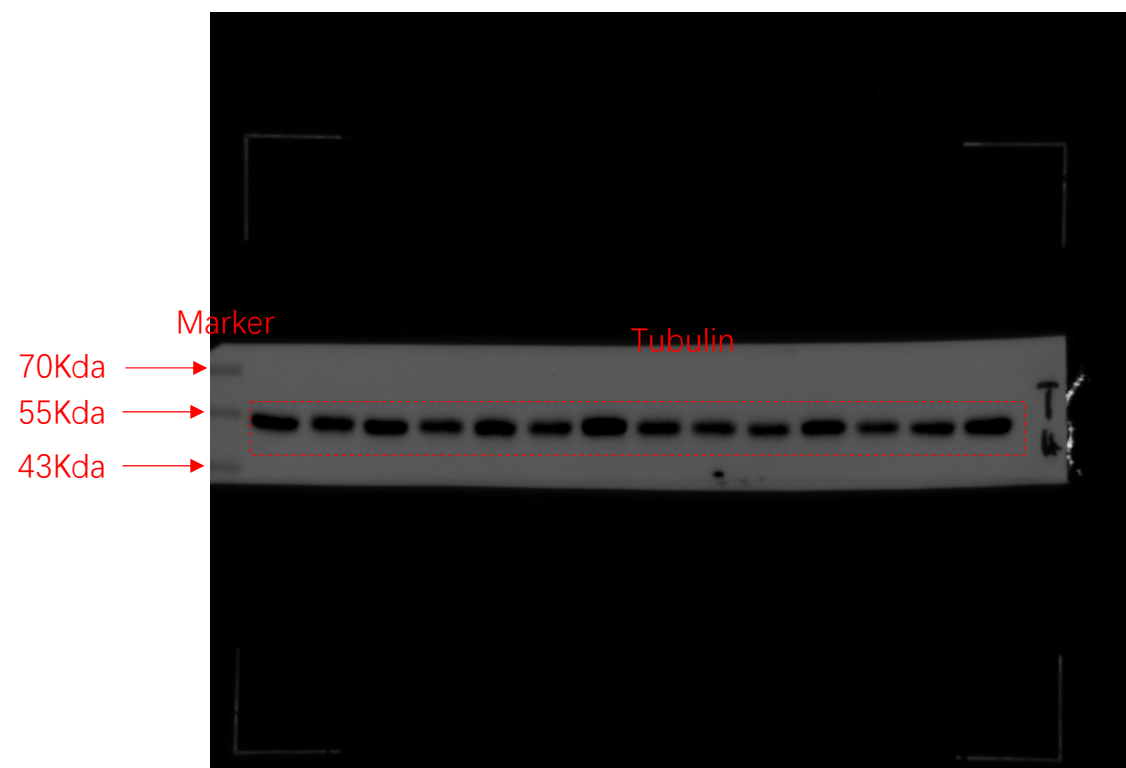

Fig 4G

SREBP1 fl (130Kda)

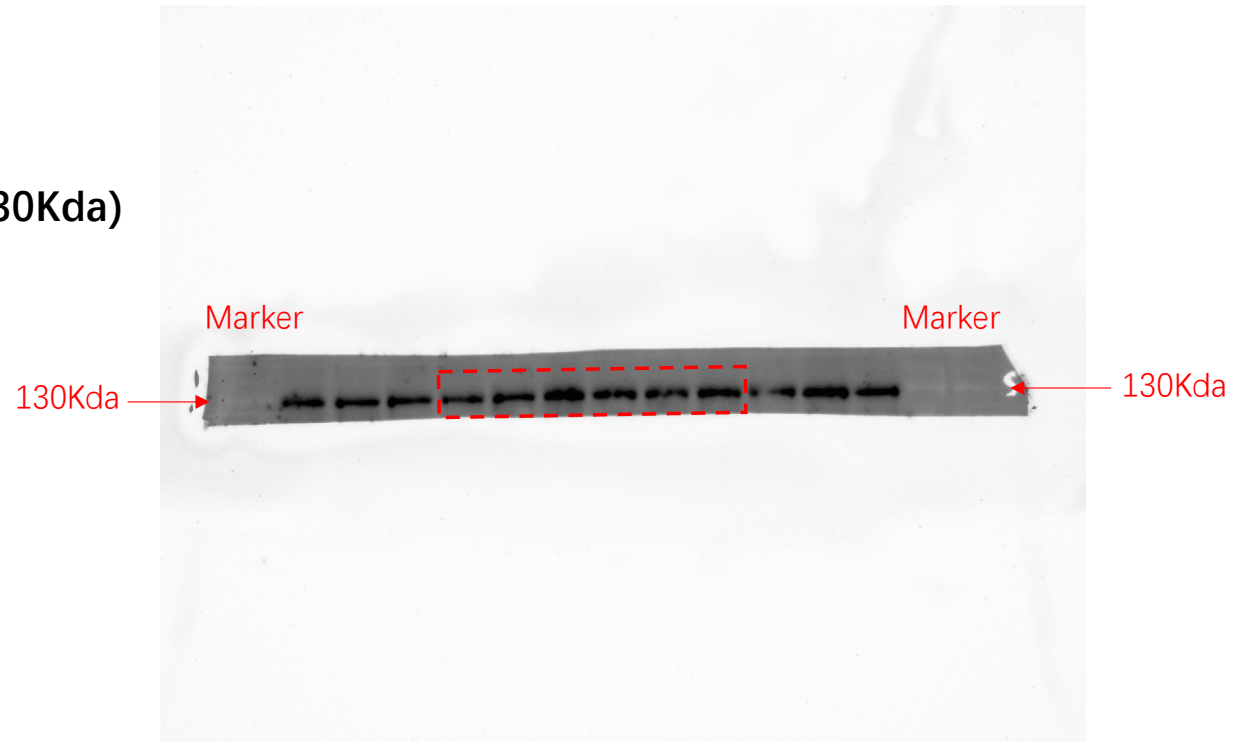

Fig 4G

SREBP1 m (68Kda)

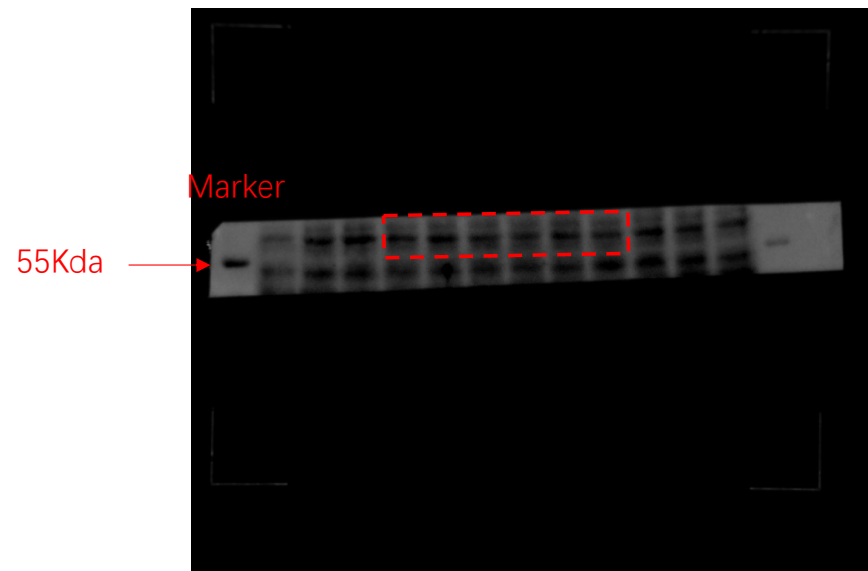

Fig 4G

SCAP

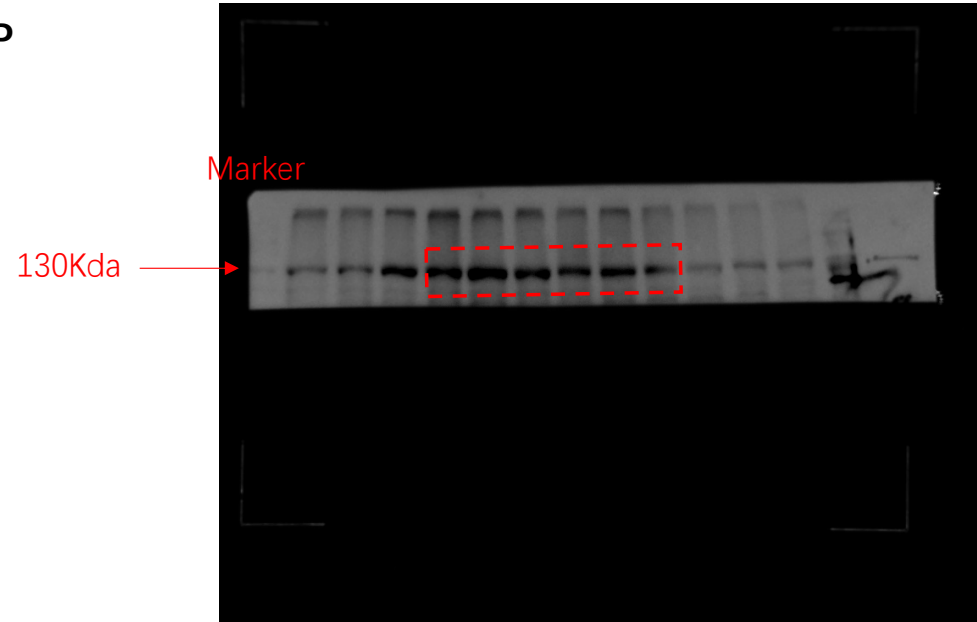

Fig 4G

Tubulin

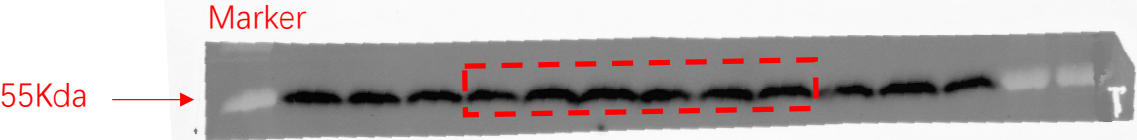

Supplement: Multimedia component 4 [file mmc4.pdf]
